# Optically Defined Modal Sensors Incorporating Spiropyran-Doped Liquid Crystals with Piezoelectric Sensors

**DOI:** 10.3390/s110201810

**Published:** 2011-01-31

**Authors:** Kuan-Ting Chen, Chin-Kai Chang, Hui-Lung Kuo, Chih-Kung Lee

**Affiliations:** 1 Engineering Science & Ocean Engineering, National Taiwan University, Taipei, 10617, Taiwan; E-Mail: ktchen@ntumems.net; 2 Industrial Technology Research Institute (ITRI), Materials & Chemical Research Laboratory, Hsinchu, 31040, Taiwan; E-Mails: ckchang@ntumems.net (C.-K.C.); HLKUO.860006@itri.org.tw (H.-L.K); 3 Institute of Applied Mechanics, National Taiwan University, Taipei, 10617, Taiwan; 4 Institute for Information Industry, Taipei, 10622, Taiwan

**Keywords:** piezoelectric, liquid crystal, spiropyran, cantilever beam, modal sensor, optically defined

## Abstract

We integrated a piezoelectric sensing layer lamina containing liquid crystals (LC) and spiropyran (SP) in a LC/SP mixture to create an optically reconfigurable modal sensor for a cantilever beam. The impedance of this LC/SP lamina was decreased by UV irradiation which constituted the underlying mechanism to modulate the voltage externally applied to the piezoelectric actuating layer. Illuminating a specific pattern onto the LC/SP lamina provided us with a way to spatially modulate the piezoelectric vibration signal. We showed that if an UV illuminated pattern matches the strain distribution of a specific mode, a piezoelectric modal sensor can be created. Since UV illumination can be changed *in situ* in real-time, our results confirm for the first time since the inception of smart sensors, that an optically tailored modal sensor can be created. Some potential applications of this type of sensor include energy harvesting devices, bio-chips, vibration sensing and actuating devices.

## 1. Introduction

Modal sensors were originally developed to eliminate a spillover problem which has its roots in noise induced by high-frequency modes in structural feedback control [1]. Modal sensors retrieve the specific modal signals of a vibration beam by matching the surface electrode shape of piezoelectric sensors to that of the strain distribution associated with respect to that mode. This technology plays an important role in achieving high-authority active control systems. The shape and electromechanical coupling coefficient of a piezoelectric material can determine the performance of a modal controller system which is typically used for active vibration control. Therefore, optimization of modal sensors is very important for different applications of active vibration control such as traditional cantilever beams, linear stochastic beams, and rectangular plates [1–3]. Continuous and array modal sensors have been designed and discussed for use in structural modal control with an attempt to pursue control of specific modes on-the-fly. Continuous modal sensors utilize the mode shape orthogonal property which matches the spatially distributed piezoelectric strength to the vibration modal strain of the structure of interest. Although model sensors have been developed for over two decades, there is still no easy way to control the spatial distribution of the piezoelectric strength [1]. The most well-known approach has been to use an effective surface electrode of the piezoelectric layer of a one-dimensional beam to tailor the spatial weighting factor associated with a one-dimensional modal strain. An array of modal sensors can be used which utilize the weighted sum of all the discrete signals measured from different locations of the structure of interest to match the specific modal strain of interest. By varying the spatial weighting factor, a different modal sensor can be created. However, spillover continues to be a problem which needs to be resolved for an array modal sensor. The modal sensor concept has been previously explored, discussed, and adopted to many different technological applications [4,5].

As mentioned above, modal sensors play an important role in active vibration control. With the rapid advancement of smart sensors/actuators, creating a methodology to enable different modal sensors in real-time has remained the primary objective over the years. A simple line of thought included spatially arranging the shape of the electrode in real-time to achieve the above-mentioned goal, however, this has remained unstudied. In our work, we developed an optical modal sensor system which integrates a lead zirconate titanate (PZT) containing liquid crystals (LC) and spiropyran (SP). This marks for the first time since the inception of smart sensors that a different modal sensor has been created in real-time. The LC/SP layer serves as the fundamental mechanism to tailor the strength of the externally applied electric field onto the piezoelectric layer achieved using only an optical method.

The liquid crystals (LC) used in this work were type E7 from Merck, and the spiropyran (SP) was from Aldrich. Recently, SP has been a popular photochromic material which has been employed in photosensitive devices due to its advantageous physical and chemical properties [6–8]. Spiropyran can be transformed into a merocyanine (MC) state under UV irradiation, and it also can be reversed to a SP state by irradiating with visible light or by heating. More specifically, the carbon-oxide bond of the spiropyran is cleaved when the spiropyran is transformed to a MC state under UV irradiation. The MC state not only converts to a polar molecule, but it also turns dark purple in color. The polar molecules provide good ionic conductivity. Spiropyran (SP) applications include sensors and transistors [9,10]. The reversion effect on such factors as dipole moment, surface energy, refractive index, and volume, enables this type of modal sensor to be used for numerous promising applications such as for actuators, modulators, optical memories, and microfluidics [11–14].

## 2. Results and Discussion

### 2.1. The Electric Properties Measurement of LC/SP

**Figure 1.**
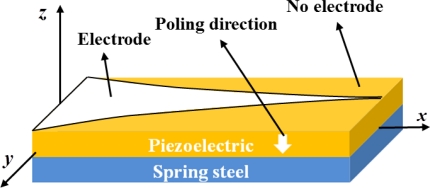
Scheme of the electrode of traditional mode 1 sensor with specific pattern employed for an one-dimensional cantilever beam.

For a one-dimensional cantilever beam, the metallic electrode of the traditional mode 1 sensor was made with a specific pattern [1]. The electrode was patterned on piezoelectric material as shown in Figure 1 and was unable to turn into a different modal sensor in real-time. The LC/SP lamina was employed to replace the traditional metallic electrode for modal sensor application due to its electrical properties. In this paper, we used LC as the solvent which was mixed with 3 wt% of the spiropyran powder. The alignment of the nematic LC (Merck E7) possessed a long-range order and its molecular shape was rod-like. The molecular shape of the doped spiropyran in the liquid crystal was also rod-like in shape. The relationship between spiropyran at small concentrations and the LC are similar to a guest-host relationship. Therefore, the doped spiropyran possessed order alignment due to the anchoring force of the liquid crystal. By incorporating a nematic LC lamina to enable the SP to retain a long-range order, the electrical properties were enhanced in a nematic orientation tailored to ion transportation [15].

**Scheme 1.**
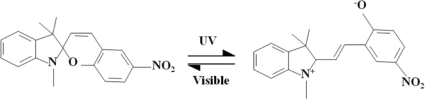
Spiropyran transformed into a MC state by UV irradiation and reversed by visible light or by heating.

Scheme 1 shows that the spiropyran can be transformed into a merocyanine (MC) state under UV irradiation, and it also can be reversed to a SP state by irradiating with visible light or by heating.

**Figure 2.**
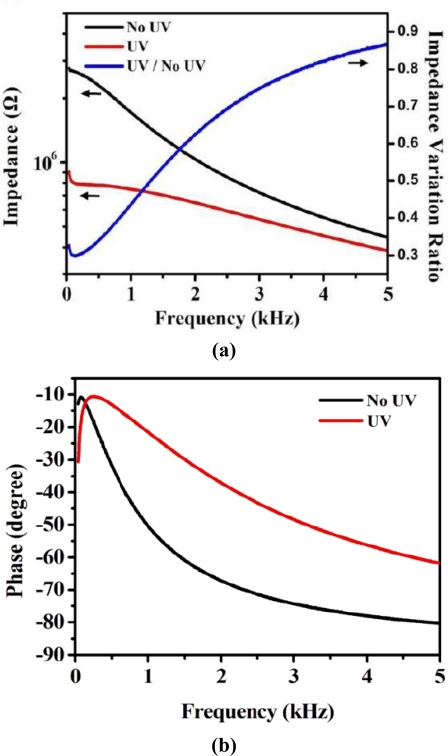
Electric properties of the LC/SP employed to replace the traditional electrode for modal sensor applications: **(a)** impedance and **(b)** phase measurements of the LC/SP under treatment of shading and UV irradiation using an impedance analyzer (Agilent 4294A).

In Scheme 1, we can see clearly that the molecular structure of the SP and MC were both rod-like in shape. Figure 2 shows the electrical properties of the LC/SP lamina measured using an impedance analyzer (Agilent 4294A). In our work, the cell was made for measuring the impedance of the LC/SP lamina.

**Figure 3.**
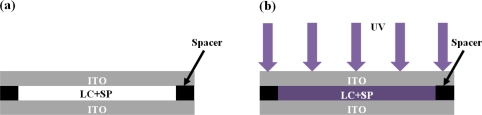
**(a)** Scheme of the cell structure composed of 200nm ITO and 20μm spacer when the LC/SP mixture was injected into the cell. **(b)** Cell schematic when sample is irradiated by UV and where the electric properties of the LC/SP mixture are measured using an impedance analyzer (Agilent 4294A).

We used a polyethylene terephthalate (PET) film coated with indium tin oxide (ITO) to enclose the LC/SP lamina (Figure 3). Rubbing the ITO surface using a velvet cloth completed the rubbing requirement to align the liquid crystal. The thickness of the PET and ITO were 188 μm and 200 nm, respectively. An adhesive containing appropriate spacers (e.g., 20 μm) was applied between the two PET+ITO plates. The LC/SP mixture was then siphoned into the cell formed by the PET/ITO plates through capillary action.

**Figure 4.**
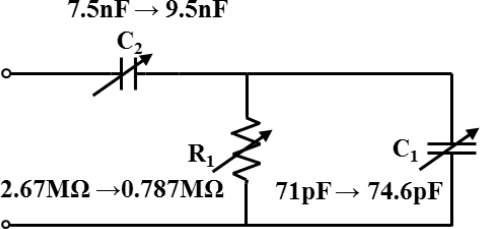
Equivalent lumped-element model of the LC/SP mixture composed of resistor (R1) and capacitors (C1, and C2). The tunable range of R1, C1 and C2 were calculated from the impedance measurement results treated without and with UV illumination.

It is clear from Figure 2(a) that the impedance of this LC/SP lamina decreased after UV irradiation. The ratio of the impedance variation represents the ratio between the impedance after and before the UV irradiation. The minimum impedance variation ratio was 0.29. The LC/SP mixture underwent 5 minutes of UV irradiation. In our experimental set-up, the wavelength and power of the UV light were 350 nm and 3 mW respectively while the distance was 2 cm between the LC/SP mixture and the UV light source. Figure 2(a) depicts the frequency response of the UV irradiated LC/SP lamina, which confirm that the resistive, capacitive and inductive properties of the LC/SP lamina varied with the UV irradiation. The variations of the LC/SP lamina impedance ratio were more pronounced at low frequency which means that the LC/SP optoelectronic material is more suitable for lower frequency applications. The phase variation shown in Figure 2(b) indicates that there is an observed higher phase shift in the higher frequency region, *i.e*., the capacitive properties of LC/SP mixture changes with UV irradiation. The less pronounced phase variation at lower frequency makes modulating the piezoelectric sensor performance for vibration control at lower frequency easier. The frequency range shown indicates that this developed material has potential application for use in active vibration control, smart sensors and actuators. Based on the electric properties of the LS/SP, the equivalent lumped-element model of the LC/SP mixture was identified to have a form of tunable resistors (R1) and capacitors (C1 and C2) as shown in Figure 4.

The tunable range of R1, C1 and C2 were calculated from the impedance measurement results. The resistance R1 was decreased from 2.67 to 0.787 MΩ and the capacitances C1 and C2 were increased from 71 to 74.6 pF and 7.5 to 9.5 nF, respectively. These results can be used to verify why the impedance and phase of the LC/SP mixture decreased under different UV exposure times. By using an equivalent lumped-element model, the impedance and phase simulation of the LC/SP mixture in Figure 3 can be obtained.

### 2.2. Electrical Properties Measurement of the LC/SP

The piezoelectric modal sensor theory developed by Lee *et al.* [1] proposed a surface electrode design by taking advantage of the orthogonality principle of mode shapes. We adopted the theory to create an optically defined modal sensor. The configuration of the mode 1 sensor is shown in Figure 1. The deformation of the y-direction was very small due to the cantilever beam geometry. We assumed there was no y-dependence in the displacement w, *i.e.*, *w* = *w*(*x,t*). In theory, the sensor equation of a single span plate can be written as:

(1) $$q(t)=-ze_{31}\int_{0}^{L}F(x)\frac{\partial^{2}w}{\partial^{2}x}dx$$

where *q*(*t*) is generated from the surface electrode of the piezoelectric lamina. Here, *F*(*x*) is assumed to be the shape of the effective surface electrode, ***e*_31_** the piezoelectric stress/charge constant, *z* the distance between the neutral axis of the beam and the middle of optical modal sensor, and *L* is the length of the beam.

**Figure 5.**
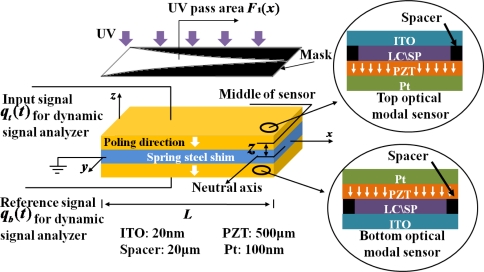
Schematic of the optical modal sensor system employed for the mode 1 sensor. The thickness of the ITO, spacer, PZT, and Pt were 200 nm, 20 μm, 500 μm, and 100 nm, respectively.

From Equation (1), the signal of the specific mode shape was obtained by changing the specific electrode. Although the impedance of the LC/SP lamina layer possessed a large variation before and after the UV irradiation, the output signal of the PZT layer not only passed through the patterned region of the electrode but also the shaded region during vibration. However, the LC/SP lamina cannot be employed directly to serve as the surface electrode of the desired modal sensor. To overcome this problem, the schematic of an optically tailored/defined optical modal sensor system was designed (see Figure 5). The optically defined modal sensor system was composed of a spring steel shim, and the top and bottom of an optical modal sensor. A LC/SP lamina was used as an electrode for the PZT layer to create the optically defined modal sensor. We removed the original Pt electrode of the PZT by using an uni-directional polish. An uni-directional scratch created from the polishing process enabled the LC/SP lamina to align well with respect to the PZT layer. As mentioned above, an adhesive containing appropriate 20 μm spacers was applied between the PZT and PET film and which was then coated with the ITO. The LC/SP lamina was then siphoned into the cavity between the PZT and ITO. The ITO was pre-rubbed with a velvet cloth. The LC/SP lamina served as the spatially modulated electric connection between the PZT and ITO. The ITO transmitted the signal from the LC/SP lamina to an external electronic instrument.

In Figure 5, we assumed that the top and bottom optical modal sensor would generate both *q_t_*(*t*) and *q_b_*(*t*) respectively. The same directional polarization of the double-layer optically defined modal sensor gave the same signal output *q_t_*(*t*) = *q_b_*(*t*) = *Q*(*t*) when the cantilever beam was excited by the shaker before UV irradiation. Both generated *Q*(*t*) without UV irradiation. The transfer function was measured using a dynamic signal analyzer (SR780, Stanford Research Systems) and the input and reference signals were provided from the top and the bottom optical modal sensors.

Before UV irradiation, the gain of the optical modal sensor system *G_system_* can be calculated as:

(2) $$G_{\mathrm{system}}=20\;{\text{log}}_{10}\left|\frac{q_{t}\;(t)}{q_{b}\;(t)}\right|=20\;{\text{log}}_{10}\left|\frac{Q(t)}{Q(t)}\right|=0$$

For the mode 1 sensor experiment, the bottom optical modal sensor was not UV irradiated. In Figure 5, we used a mask with a specific pattern of a modal sensor to irradiate at specific locations on the top optical modal sensor with UV light. As the UV irradiated region at the top optical modal sensor possessed a low LC/SP lamina impedance, more PZT output signals were generated in this region. The top optical modal sensor was assumed to have generated the signal:

(3) $$q_{t}(t)=Q(t)+q_{\mathrm{eff}}$$

where *q_eff_* was the assumed shape of the effective surface electrode defined by UV irradiation. The total signals then contributed by *Q*(*t*) and by UV effect can be obtained. From Equations (1,3), the gain can be written as:

(4) $$G_{\mathrm{system}}=20\;{\text{log}}_{10}\left|\frac{q_{t}\;(t)}{q_{b}\;(t)}\right|=20\;{\text{log}}_{10}\left|1+\frac{-ze_{31}\int_{0}^{L}F_{1}(x)\frac{\partial^{2}w}{\partial^{2}x}dx}{Q(t)}\right|$$

Here, *F_1_*(*x*) is the shape of the effective surface electrode of mode 1 sensor. From Equation (3), the mode 1 sensor was be obtained by using this optically defined modal sensor system.

**Figure 6.**
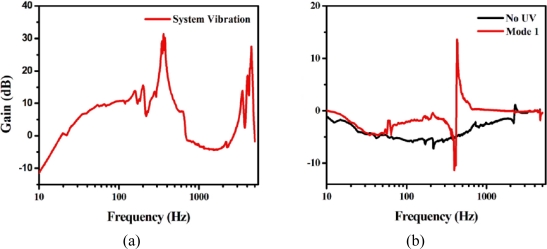
**(a)** The eigen-frequency of the vibration of optical modal sensor system measured from the top optical modal sensor by using a dynamic signal analyzer (SR780). **(b)** Transfer function of the optically defined modal sensor system treated without and with specific patterns as measured by the SR780.

The eigen-frequency of the vibration of the optically defined modal sensor system was measured from the top sensor by using a dynamic signal analyzer (SR780) [see Figure 6(a)]. The natural frequencies of mode 1 and mode 2 of the cantilever beam were 361 Hz and 4,532 Hz, respectively. Figure 6(b) experimental result shows signals of mode 1 and mode 2 which were obtained independently without UV irradiation. The specific pattern that was employed for the mode 1 sensor was made under UV irradiation which allowed only the mode 1 signal to prevail. The natural frequencies of 427.05 Hz are shown in Figure 6(b). The natural frequency of mode 1 shifted from 361 to 427.05 Hz after the UV irradiation, and can be attributed to the capacitive and inductive properties variation of the LC/SP lamina.

## 3. Conclusions

In summary, the experimental results demonstrate that the electric properties of our LC/SP lamina are affected by UV irradiation. In our configuration, the large impedance variation of the LC/SP lamina was enough to modulate the PZT sensor signal. The results also show that mode 1 can be successfully created by making a specific pattern and that the optical modal sensor can be controlled by UV irradiation. Therefore, our optical modal sensor can be utilized to detect other vibration modes by illuminating with other specific patterns. The experimental results also reveal a method for creating optically defined modal sensors.
